# Age and Perceived Employability as Moderators of Job Insecurity and Job Satisfaction: A Moderated Moderation Model

**DOI:** 10.3389/fpsyg.2019.00799

**Published:** 2019-04-12

**Authors:** Jesus Yeves, Mariana Bargsted, Lorna Cortes, Cristobal Merino, Gabriela Cavada

**Affiliations:** School of Psychology, Universidad Adolfo Ibáñez, Santiago, Chile

**Keywords:** job insecurity, perceived employability, job satisfaction, age, extrinsic satisfaction, intrinsic satisfaction

## Abstract

Workforce ageing and the need to work longer implies several challenges worldwide. Due to the potential for career prolongation, one such implication is to understand how age and perceived employability buffers relationship effects between job insecurity and job satisfaction. Therefore, this research aims to investigate the moderating roles of perceived employability and age on the relationship between job insecurity and job satisfaction. Hypotheses were tested using a three-way interaction model based on a cross-sectional design with a representative sample of 1,116 Chilean workers. Results show that age plays an important role in employees with high perceived employability; however, it has no effect on employees with low perceived employability. Younger workers with high perceived employability suffer less than do older employees with high perceived employability in terms of intrinsic job satisfaction. From a theoretical point of view, perceived employability in older workers does not reduce the unfavorable consequences of job insecurity. Regarding practical implications, organizations should manage and develop older workers by focusing on intrinsic aspects of their careers and on retirement preparation, as this will improve control and other positive resources in this population.

## Introduction

Over the last quarter of the 20th century and the first decades of the 21st, there has been a radical shift in working environments from secure to insecure due to unprecedented transformations in demography, labor market, technology, economy, and politics. Insecurity regarding the future of work is demonstrably increased (Benach et al., 2014), and many factors compound the risk of job insecurity, e.g., high-to-low shifts in mortality and fertility, which have accelerated the ageing of world populations (Andreev et al., 2013).

Job insecurity has been shown to impact individual behavior, well-being, and work attitudes (Sverke et al., 2006). Negative consequences of job insecurity include, for instance, increased burnout (Aybas et al., 2015), decreased well-being (Berntson et al., 2010; Green, 2011), and life satisfaction (Silla et al., 2009; Sora et al., 2010). Job insecurity has also been shown to be related with overall job satisfaction (De Cuyper et al., 2009), and also with intrinsic and extrinsic job satisfaction (Buitendach and De Witte, 2005). The present research focuses on the above relationship, testing the moderating role perceived employability and age play in this relationship.

Of great interest to the field, then, would be a better understanding of concepts associated with ameliorating the effects of job insecurity, known as “buffer mechanisms.” Employability has been studied as one such mechanism, as have age differences. In the former, it is important to take a more in-depth look at the perceived aspects of being employable, since self-perception influences behavior, reactions, and thoughts within professional lives and beyond (Berntson, 2008). For the latter, what little has been studied on the moderating effect of age on the relationship between job insecurity and job satisfaction is inconsistent: as Lee et al. (2018) established, it is important to consider how demographic differences influence individual responses to job insecurity. From a job dependence perspective (Greenhalgh and Rosenblatt, 1984), older workers are more vulnerable to experience job insecurity because they are more dependent on their current jobs (Cheng and Chan, 2008). In addition, older employees tend to perceive themselves as less employable when comparing themselves to younger counterparts (Van der Heijden, 2002; Rothwell and Arnold, 2007; Wittekind et al., 2010; Froehlich et al., 2015; Peeters et al., 2016). Regarding both age and employability, older and younger workers may cope differently with either the consequences or the negative stereotypes related to older workers depending on how they perceive their employability (Posthuma and Campion, 2008; Finkelstein et al., 2015). Indeed, age can have negative consequences on job offers for older workers (Ahmed et al., 2012). Some job dimensions, such as high learning value, might affect self-reported employability, which may be moderated by age (Van der Heijden et al., 2016). Previous studies have also predominantly indicated that young individuals self-report higher employability over the elders (Van der Heijden, 2002; Van der Heijde and Van der Heijden, 2006; Rothwell and Arnold, 2007; Wittekind et al., 2010; Froehlich et al., 2015; Peeters et al., 2016; Böttcher et al., 2018). Indeed, several studies have shown a negative relationship between age and perceived employability (Van der Heijden, 2002; Van der Heijde and Van der Heijden, 2006).

Given the conditions of job insecurity as above, it is clear that society must redouble its efforts to maintain job satisfaction. Understanding how both intrinsic and extrinsic satisfaction are affected by job insecurity, and how this can be moderated by age, employability, or a combination of these. The latter is important and is the focus of this paper. As Froehlich et al. (2015) affirm, identifying how perceived employability influences the relationship between job insecurity and job satisfaction across different career stages is critical to developing counseling interventions and policy recommendations.

As such, this research seeks first, to examine the effects of job insecurity on job satisfaction (extrinsic and intrinsic); and second, how perceived employability and age moderate that relationship. The practical implications are clear. By exploring these novel three-way interactions moderating both intrinsic and extrinsic job satisfaction, this contribution to the field of job insecurity will aid in coping with the increasing tension between labor instability and the ageing population. Therefore, getting older could be a resource or a demand depending on context variables, becoming the real threat that could interact with employability. Understanding the role age could play in coping with job insecurity, could help develop specific career interventions, aiming to address vulnerability and discrimination older workers may suffer, who, having employability, are forced to finish their careers prematurely.

### Job Insecurity and Job Satisfaction

The term job insecurity has been used in a variety of ways. The current paper defines job insecurity as “the subjectively perceived and undesired possibility to lose the present job in the future, as well as the fear or worries related to this possibility of job loss,” following Vander Elst et al. (2014, p. 365). Within this perspective, job insecurity not only denotes perceived threats to the job as a whole (De Witte, 1999), but has also been shown in the literature to influence several attitudinal outcomes (Lee et al., 2018). In a recent meta-analysis, Sverke et al. (2018) stated that the relationship between job insecurity and several other organizational variables is strong and stable over time; however, there is still no evidence of intervening variables.

As such, the present paper focuses on the means through which job insecurity impacts job satisfaction, which is considered a “key attitudinal outcome,” with a particular emphasis on both intrinsic and extrinsic aspects. This distinction is not irrelevant; rather, job satisfaction can be affected specifically in either category rather than a more general approach (Spector, 1997; Gamboa et al., 2009), and this two-dimensional approach to job satisfaction allows further comprehension of both components (Oldham and Hackman, 2010) to interrelate differently with other variables, e.g., job involvement (Hirschfeld, 2000). Job satisfaction mediates the relationship between job insecurity and other aspects, such as organizational citizenship behavior, deviant behavior, anxiety, anger, and burnout (Reisel et al., 2010); exit, voice, loyalty, and neglect (EVLN) responses (Tayfur et al., 2018); life satisfaction of family members (Emanuel et al., 2018); and Decent Work, specifically as measuring intervention impact on reducing the negative effects of job insecurity (Emanuel et al., 2018; Tayfur et al., 2018; Masdonati et al., 2019). As such, job satisfaction is relevant in understanding the relationship among all these constructs and has been shown to moderate some negative consequences of job insecurity.

Several theories have attempted to explain the above relationships among various aspects of job insecurity and job satisfaction. These include stress theory, job demands-resources model, and psychological contract theory. According to stress theory (Lazarus and Folkman, 1984), job insecurity is perceived as a work stressor, given that the anticipation of possible job loss can be perceived as a source of anxiety as importantly as the loss itself. Cheng and Chan (2008) pointed out that job insecurity is a powerful stressor, which results in a variety of strain outcomes, including poor well-being, diminished job attitudes, and poor performance. Next, according to the job demands-resources model (Bakker and Demerouti, 2007, 2013), job insecurity can be seen as a threatening demand, in particular, when demands are higher than the resources (provided by the organization or personal ones); or when the existing resources are not enough to buffer the impact of job demands on strain, resulting in a decrease of job satisfaction. Third, psychological contract theory (De Cuyper and De Witte, 2006) suggests that perceived violations of socio-emotional expectations are manifested as job insecurity, i.e., the disconnect between employee effort and employer security; that situation may lead employees to decrease loyalty and effort in order to restore the balance of the expectations between employers and employees (Vander Elst et al., 2014). Regardless of framework, it can be expected that job insecurity has negative outcomes.

Indeed, a growing body of research based on these theories has pointed out that job insecurity negatively affects employee attitudes, behaviors, and health (Sverke et al., 2002; Cheng and Chan, 2008; Silla et al., 2009; Sora et al., 2010; Peiró et al., 2012). Moreover, consistent evidence indicates negative associations among job insecurity and key attitudinal outcomes, such as employee organizational commitment and job satisfaction (Ito and Brotheridge, 2007; De Cuyper et al., 2009; König et al., 2011; Debus et al., 2012; Jiang and Probst, 2016). Interestingly, between extrinsic and intrinsic job satisfaction, Buitendach and De Witte (2005) found that job insecurity negatively impacts extrinsic, but not intrinsic, job satisfaction; this suggests that job insecurity is related with extrinsic aspects of job satisfaction, such as the type of contract. However, considering that job insecurity has a negative impact on wellbeing (Mauno and Kinnunen, 1999) it is worth reviewing if intrinsic job satisfaction is also negatively impacted.

Based on these theoretical frameworks and on the results of previous studies, the first hypothesis was formulated:

Hypothesis 1: Job insecurity will negatively correlate with intrinsic (H1a) and extrinsic (H1b) job satisfaction. The relationship will be stronger for H1b than for H1a.

### Perceived Employability as Moderator

There is no consensus on how to define employability (Kluytmans and Ott, 1999; Fugate et al., 2004). However, authors generally agree that employability refers to employee ability to obtain and maintain employment (Hillage and Pollard, 1998); to the chances of finding alternative employment, either on the internal or the external labor market (Forrier and Sels, 2003); and to the development of variegated attributes, abilities, and competences. In the latter sense, Fugate et al. (2004) conceptualized employability as “a form of work-specific active adaptability that enables workers to identify and realize career opportunities” (p. 16), composed of the following three dimensions: human and social capital, career identity, and personal adaptability. Similarly, Van der Heijde and Van der Heijden (2006) considered employability to be “the continuous fulfilling, acquiring or creating of work through the optimal use of competences” (p. 453), which establishes a model of employability based on occupational expertise, anticipation and optimization, personal flexibility, corporate sense, and balance. From a subjective perspective, perceived employability was defined as “the individual’s perception of his or her possibilities of getting new employment and is thus approached from the individual’s perspective” (Berntson and Marklund, 2007, p. 281). Employability is related to the perception one has toward the opportunities available and the amount of effort needed to get a job.

In all, employability represents how attractive individuals can be to the labor market, where highly employable people are more valued by employers (Berntson et al., 2006). Moreover, employable people tend to seek better jobs; they are more likely to quit jobs they find unrewarding or unsatisfying; and act upon their perception that there are better alternatives, particularly by engaging in job search behavior (De Cuyper et al., 2008). McArdle et al. (2007) showed how highly employable individuals have better outcomes at finding new jobs than those with lower employability levels. Indeed, those with high perceived employability usually receive more job offers (De Cuyper et al., 2009; Silla et al., 2009) and have more opportunities to leave low quality jobs for better ones (Berntson and Marklund, 2007).

Although job insecurity and perceived employability are comparable in that they are subjective perceptions regarding the future (De Witte et al., 2015), there is an important distinction: job insecurity relates to the future of the current job, whereas perceived employability concerns future jobs – be these with the current employer or another (De Cuyper et al., 2018). De Witte and De Cuyper (2015) summarized the relationship between job insecurity and perceived employability from two points of view. First, job insecurity might mediate perceived employability in regards to several outcomes. For example, more employable people may perceive less job insecurity, which may lead them to feel less exhausted (De Cuyper et al., 2012). Perceived employability among workers who experience job insecurity increases risk of deviant behaviors and intentions to leave; in such conditions, employees are likely to develop rationalizations to decrease cognitive dissonance regarding their moral disengagement (De Cuyper et al., 2009; Huang et al., 2017). Second, over the past few years, there has been increasing research into perceived employability as moderator of relationships among perceived job insecurity and its negative consequences. Karasek (1979) also sought to explain that interaction with job demands-control model, which considers job insecurity as highly demanding, and perceived employability as highly controlled; in that model, a combination of high demands and low control leads to employment strain, increasing job dissatisfaction. In addition, the interaction between job insecurity and employability, can be explained by the job demand resources model (Demerouti et al., 2001). The model states that resources act as buffers against the negative “job strain” effects that job demands produce. Consequently, many authors suggest that resources are the most important predictors of job satisfaction (Demerouti et al., 2001; Bakker and Demerouti, 2007, 2013). In this sense, perceived employability can be modelled as a personal resource that increases job satisfaction by buffering the negative effects of job insecurity (as a demand). When perceived employability is high, the worker sense of control increases, leading to development of proactive behaviors to deal with job insecurity (Lee et al., 2018). Empirical evidence has since shown how perceived employability buffers the negative effects of job insecurity (De Cuyper et al., 2008). For example, Aybas et al. (2015) found that perceived employability buffers the negative relationship between job insecurity and burnout; Silla et al. (2009) found that the negative effects of job insecurity on life satisfaction were significantly reduced in employees with high perceived employability, with a special emphasis on how perceived employability helps employees to cope with their perceptions of job insecurity; and still other studies have supported the interaction between job insecurity and perceived employability in relation to well-being (Berntson et al., 2010; Green, 2011).

Regarding the effect that perceived employability exerts on job satisfaction, several authors have shown a positive relationship between both variables (Hillage and Pollard, 1998; Forrier and Sels, 2003; Berntson and Marklund, 2007; De Cuyper et al., 2008). Though distinguishing between measures of extrinsic and intrinsic job satisfaction, Gamboa et al. (2009) found positive relationships between perceived employability and both dimensions of job satisfaction. Furthermore, perceived employability has been explored for intrinsic and extrinsic job aspects (Van Emmerik et al., 2012): for intrinsic aspects, worker sense of competence improves their perceived employability; while extrinsic aspects relate to perceived employability via the perception of, for example, upward mobility (perceiving more opportunities to achieve different tangible rewards).

The aforementioned job demands-resources model, in which job insecurity as job demand can affect extrinsic and intrinsic dissatisfaction, while perceived employability as personal resource can affect higher intrinsic and extrinsic levels of satisfaction (Lu et al., 2015; De Cuyper et al., 2018), has led to the following hypothesis:

Hypothesis 2: Perceived employability will moderate the relationship between job insecurity and intrinsic (H2a) and extrinsic (H2b) job satisfaction. The negative relationship between job insecurity and job satisfaction will be weaker under the condition of high versus low perceived employability.

### Age as Moderator

As a demographic factor, age has the potential to affect job insecurity and its relationship with job satisfaction. Though empirical evidence on the relationship between age and job insecurity is not entirely clear, some studies have shown that younger people experience higher levels of job insecurity (Roskies and Louis-Guerin, 1990; Keim et al., 2014). However, other studies have reported that older people experience higher levels of job insecurity (Mohr, 2000; Näswall and De Witte, 2003). Interestingly, Fullerton and Wallace (2007) identified a curvilinear relation between age and job insecurity.

The impact of age on the relationship between job insecurity and job satisfaction might be influenced by age stereotypes: according to Posthuma and Campion (2008), negative consequences of age-related stereotypes include lowered motivation, capacities, productivity, sense of belonging, and higher difficulties in learning and resistance to change. Stereotypes regarding older people are different in each life domain (Kornadt and Rothermund, 2011), and have been consistently refuted in the work domain (Posthuma and Campion, 2008). Even so, when these stereotypes are negative, they can have adverse effects on self-esteem, encourage discrimination, and even self-exclusion (Weiss et al., 2013). Meta-stereotypes can also play a role within this dynamic. Meta-stereotypes are beliefs a person has about the stereotypes that others, typically individuals outside the group, have of them; these assumptions vary according to the outside reference group (Vorauer et al., 1998). Meta-stereotypes can be classified as positive or negative, and they result in several reactions. For example, a positive meta-stereotype can activate emotions such as happiness and satisfaction or may lead to a sense of threat; a negative-stereotype may also be perceived as a threat, or as an opportunity and a source of motivation (Finkelstein et al., 2015). However, negative meta-stereotypes are more likely to negatively affect perceived employability (Owuamalam and Zagefka, 2014; Finkelstein et al., 2015). Finkelstein et al. (2013) found that older workers focus mainly on negative characteristics, tending to believe younger people view them as boring, old, stubborn, and grumpy. Although these meta-stereotypes were proven not to be accurate, even so, younger workers, described them as responsible, mature, and hard working. Notwithstanding that older workers thought middle-aged workers see them less negatively than younger people, stereotypes were actually more positive than what they expected.

Older workers may therefore respond more strongly to job insecurity than younger employees, since they may be more sensitive to economic insecurity or more dependent on their current jobs (Greenhalgh and Rosenblatt, 1984), which may be taken as a threat to their work identity (Cheng and Chan, 2008). Claes and Van De Ven (2008) compared the relationship between job insecurity and job satisfaction among younger employees (less than 25 years old) and older workers (more than 50) and found that the negative effect of high job insecurity on job satisfaction was buffered for younger workers. However, a meta-analysis by Cheng and Chan (2008) found the association between job insecurity and job satisfaction not to differ between younger and older employees. Additionally, there is some evidence arising from studies into the relationship between age and job satisfaction. Clark et al. (1996) reported a U-shape relationship between age and overall satisfaction, satisfaction with pay (extrinsic job satisfaction) and satisfaction with work itself (intrinsic job satisfaction). Also, Ng and Feldman (2010), in a meta-analytic review, found a positive, though weak, relationship between age and overall, intrinsic, and extrinsic job satisfactions. However, when controlling for organizational tenure, the relationship between age and intrinsic satisfaction increased, and the relationship between age and overall and extrinsic satisfaction decreased. Moreover, Drabe et al. (2015) found that both intrinsic and extrinsic job characteristics are more predictive of younger employee job satisfaction, while job satisfaction for older employees is related to establishing good colleague relationships. Finally, the impact of age on the relationship between job insecurity and job satisfaction may be higher for older workers when job insecurity is related to retirement concerns (Gaines et al., 2018).

As noted, the relationship between job insecurity and job satisfaction may be weaker for younger workers than for older workers, especially given the latter’s sensitivity to economic insecurity, dependence on their positions, and their proximity to retirement. Considering these arguments, the third hypothesis of this study is:

Hypothesis 3: Age will moderate the relationship between job insecurity and intrinsic (H3a) and extrinsic (H3b) job satisfaction. The negative relationship between job insecurity and job satisfaction will be weaker for younger employees than for older employees.

### The Interaction Between Perceived Employability and Age

While acknowledging that the above hypotheses may explain some variance in the complex interactions among job insecurity, age, and perceived employability, it is important to fully explore the relationships among variables. Indeed, several studies have attempted to describe the relationship between age, perceived employability, stereotypes, and meta-stereotypes (Ahmed et al., 2012; Finkelstein et al., 2013; Van Selm and Van der Heijden, 2013). Of particular interest, Froehlich et al. (2015) proposed a model for not just chronological age, but also future time perspective and goal orientations, to understand how age can interact with employability. Though the authors conclude a positive relationship between formal learning at work and employability, they nonetheless suggest a negative relationship between ageing and formal learning; as workers get older, they lose interest in formal learning, potentially resulting in older workers missing opportunities to enhance employability through training. Similarly, Truxillo et al. (2015) state that older workers have increased interest in the intrinsic aspects of work, such as accomplishment, connection with others, autonomy, and social motives, but decreased interest in growth motives (achievement and mastery), which are some of the aspects most valued by the labor market.

The job demands-resources model is thus a useful framework for integrating all the variables under study. Age may be conceptualized as a demand for older workers, given the negative outcomes at work stemming from meta-stereotypes (Finkelstein et al., 2013). At the same time, age would be a resource for young workers, due to the positive perception older worker groups share of them, perceiving young employees as talent that needs to be encouraged and developed (Finkelstein et al., 2013). Ageing is a normative and unavoidable process, it is not something people have control over, so as workers get older, the meta-stereotypes on how younger workers view them, may become more negative. The job demands-resources model guides this complex interplay as a very clear increase in demands, resulting in a perception of loss of control. Based on this line of reasoning on meta-stereotypes, it is likely harder for older workers to cope with job insecurity.

It has been observed that differences in age result in differences of cognition, personality, and motivational changes, which derive in that the efforts made to achieve good performance levels differ among different age groups (Truxillo et al., 2015). In addition, Truxillo et al. (2015) found that age correlates both with increased crystallized intelligence (implying greater wisdom, knowledge, and skills) and with decreases in fluid intelligence (processing speed, working memory, and selective attention). In this sense, Guglielmi et al. (2016) stated that older workers need more effort to achieve higher levels of work performance when facing tasks based on fluid intelligence. From the labor market perspective, the new resources gained when ageing (crystallized intelligence) are not valued; rather, the decrease in certain skills (fluid intelligence) might lead older workers to perceive themselves as less valued by the environment. Therefore, in addition to meta-stereotypes, this paper considers age a demand for fluid intelligence tasks for older workers, and a resource for younger workers given the same situation. Indeed, the meta-analysis by Bal et al. (2011) examined age effects on several organizational decisions and found that higher age was associated with negative outcomes regarding advancement, overall evaluation, and selection.

As noted, employability may be conceptualized as a personal resource that addresses job insecurity (demand). Given the situation of young employees with high perceived employability, workers hold two resources (age and perceived employability) that interact to increase the perception of control and optimism in coping with job insecurity. Contrarily, an older worker with high perceived employability will have only the resource of employability, and thus greater demand (job insecurity and age). This is likely compounded by a perception of less opportunity following job loss, which would further weaken employability as a resource; and further by the loss of perceived control in the presence of relevant macro-level factors like retirement, downsizing, or corporate culture (Schreurs et al., 2011). Therefore, age is a relevant variable in work contexts, and since negative meta-stereotypes regarding age are not uncommon (Finkelstein et al., 2015), any increased negative reaction to meta-stereotypes would easily counteract the positive effects employability has on job insecurity and job satisfaction.

Based on the preceding information, the fourth hypothesis is:

Hypothesis 4: There will be a three-way interaction between job insecurity, perceived employability, and age in the prediction of intrinsic (H4a) and extrinsic (H4b) job satisfaction. Young employees who perceive job insecurity and have higher perceived employability will have higher job satisfaction.

## Materials and Methods

### Sample

This study uses data from the 2018 national panel study, *Zoom al trabajo* (a closer look at work, in English), annually conducted since 2014 through personal interviews. The 2018 iteration worked with 1,586 Chileans with paid jobs, from 18 to 80 years old, living in the thirteen largest cities of the country. This study makes use of probabilistic sampling, and results are weighted by sex, age, socioeconomic status, and city size. The sampling error was 2.5%. Of the interviewees, 29.6% were excluded for the purposes of this study because they were self-employed, and so did not fit criteria to answer the questions regarding job satisfaction. The effective sample was thus *N* = 1,116, with 56.5% of the respondents male. The age composition of the sample was as follows: 22.2% were 18–29; 36.7% were 30–44; 34.9%, 45–59; and 6.2%, 60–65 years old. The average age was 40.21 years old (*SD* = 11.68). The distribution of socioeconomic status^1^ is as follows: 1.6% AB; 16.6% C1A; 14.8% C1B; 26.2% C2; 24.2% C3; and 16.7% D.

### Measures

Analyses were controlled for gender (1 = men; 0 = women), socioeconomic status (1 = D; 2 = C3; 3 = C2; 4 = C1B; 5 = C1A; 6 = AB), and formality of employment (1 = formal employment; 0 = informal employment). The choice of control variables was based on previous research findings, which indicated that men tend to report better perceived employability (Flecker et al., 1998), as well as higher levels of job insecurity (Kinnunen et al., 2000) compared with women. Also, Sloane and Williams (2000) found that women are more satisfied with their jobs than men. Regarding to socioeconomic status, Wunder and Schwarze (2006) stated that income inequality has an impact on individual welfare. In addition, labor market participation of ageing workers in Chile is characterized by jobs that tend to exhibit greater levels of informality and precariousness (Vives et al., 2016). In this avenue, Cassar (2010) found that informal employees in Chile tend to have poorer job protection than do formal workers, which lowers job satisfaction.

Job insecurity was measured using the Job Insecurity Scale (JIS), developed by De Witte (2000) (see Vander Elst et al., 2014). This scale is composed of four items (5-point Likert scale), and has been validated in English, Spanish, Dutch, Flemish, and Swedish. The Spanish version had an internal consistency of 0.87. In this study, Cronbach’s alpha was also 0.87. The scale evaluates the threat of job loss and concerns about job loss. An item example is “Chances are, I will soon lose my job.”

### Age Was Measured in Years

Perceived employability was measured using a 5-point Likert scale of four items, developed by De Witte (1992) (see De Cuyper et al., 2014), which has an internal consistency of 0.92, and a Cronbach’s alpha between 0.85 and 0.90 (Van Hootegem et al., 2018). In this study, internal consistency was 0.91. The items were “I could easily find another job, if I wanted to”; “I am confident that I could quickly get another job”; “I am optimistic that I would find another job, if I looked for one”; and “I will easily find another job if I leave this job.”

Measures of job satisfaction were based on García-Montalvo et al. (2003), who described extrinsic satisfaction as an emotional response or general attitude toward extrinsic aspects of the labor activity itself, such as economic resources, stability at work, promotion opportunities, or labor conditions; and intrinsic satisfaction, as an emotional response to aspects of the job itself, such as opportunities to learn, the variety of tasks and the skills required for a given job, and autonomy. The measure takes nine items with a 7-point Likert scale: intrinsic job satisfaction was measured using five items referring to development and training opportunities, voice and participation opportunities, enjoyment, space for creativity and innovation, and autonomy to make decisions; and the extrinsic job satisfaction dimension was measured with items on income and rewards, job stability, work-non-work conciliation, and organizational prestige. Internal consistency of intrinsic job satisfaction was 0.89, and for extrinsic 0.75.

### Analytical Procedure

Hierarchical regression analyses were used to test hypothesized relationships. To alleviate the potential multi-collinearity problem, variables were mean-centered before creating the interaction terms (Aiken and West, 1991). Hierarchical regression in this study had four successive steps. The first step included the control variables; the second step, effects of job insecurity, perceived employability, and age; the third, three cross-product terms for job insecurity and perceived employability, job insecurity and age, and perceived employability and age; and finally, the cross-product terms of the three predictors in order to test hypothesized interaction effects. For a more specific test of the hypotheses, a simple slope analysis was conducted following Aiken and West (1991). Slope difference tests were also calculated following Dawson and Richter (2006).

## Results

The means, standard deviations, reliabilities, and correlations among key variables are shown in Table 1.

**Table 1:** Descriptive statistics (means and standard deviations) and correlations.

Variables	*M*	*SD*	1	2	3	4	5	6	7	8
(1) Gender (1 = male)	-	-	-							
(2) Socioeconomic state	2.24	1.32	0.14***							
(3) Formal employment (1 = yes)	-	-	0.01	0.06*						
(4) Perceived employability	3.35	0.94	0.06*	-0.10**	0.02	(0.91)				
(5) Job insecurity	2.67	0.90	0.03	-0.18***	-0.13***	-0.14***	(0.87)			
(6) Age	40.21	11.68	0.19***	0.28***	0.09***	-0.24***	-0.02			
(7) Intrinsic job satisfaction	4.92	1.22	0.01	0.05	-0.03	0.18***	-0.30***	0.02	(0.89)	
(8) Extrinsic job satisfaction	5.25	1.13	0.01	0.05	0.03	0.20***	-0.40***	0.04	0.75***	(0.75)


*Cronbach α in brackets ^∗^*p* < 0.05; ^∗∗^*p* < 0.01; ^∗∗∗^*p* < 0.001.*

Hypothesis 1 posited that job insecurity would be negatively related to intrinsic and extrinsic job satisfaction. The results obtained by hierarchical multiple regression (see Table 2), after controlling variables at step 1, indicated that job insecurity was negatively related to intrinsic (β = -0.28; *p* < 0.001) and extrinsic job satisfaction (β = -0.38; *p* < 0.001). Therefore, these results support hypotheses 1a and 1b. In addition, perceived employability and age were positively related to intrinsic (perceived employability: β = 0.15; *p* < 0.001; age: β = 0.06; *p* < 0.05) and extrinsic job satisfaction (perceived employability: β = 0.17; *p* < 0.001; age: β = 0.08; *p* < 0.05). This step increased the explained variance of job satisfaction indicators (intrinsic: Δ*R*^2^= 0.115, *p* < 0.001; extrinsic: Δ*R*^2^= 0.182, *p* < 0.001).

**Table 2:** Hierarchical regression analysis of job insecurity, employability, and age in predicting intrinsic and extrinsic job satisfaction.

	Intrinsic job satisfaction				Extrinsic job satisfaction			
		
	Step 1	Step 2	Step 3	Step 4	Step 1	Step 2	Step 3	Step 4
***Step 1: Control variables***		
Gender (1 = male)	0.01	0.01	0.01	0.01	0.01	0.01	0.01	0.01
Formal employment (1 = yes)	-0.03	-0.08**	-0.07*	-0.07*	0.03	-0.03	-0.03	-0.03
Socioeconomic state	0.05	0.00	0.00	0.00	0.05	-0.02	-0.02	-0.02
***Step 2***		
Perceived employability		0.16***	0.16***	0.16***		0.17***	0.18***	0.18***
Job Insecurity		-0.28***	-0.29***	-0.30***		-0.38***	-0.38***	-0.39***
Age		0.06*	0.05	0.04		0.08*	0.08*	0.08*
***Step 3: Interactions***		
Job Insec × Employa			-0.01	0.00			0.06*	0.07*
Job Insec × Age			-0.08**	-0.08**			-0.03	-0.03
Employa × Age			-0.05*	-0.06*			-0.04	-0.05
*Step 4:*		
Job Insec × Employa × Age				-0.07*				-0.04
*R*^2^	0.003	0.118***	0.125***	0.129***	0.003	0.186***	0.192***	0.194***
Δ*R*^2^		0.115***	0.007*	0.004*		0.182***	0.007*	0.002


*^∗^*p <* 0.05; ^∗∗^*p* < 0.01; ^∗∗∗^*p* < 0.001. Insec = Insecurity; Employa = Employability*.

**FIGURE 1:**
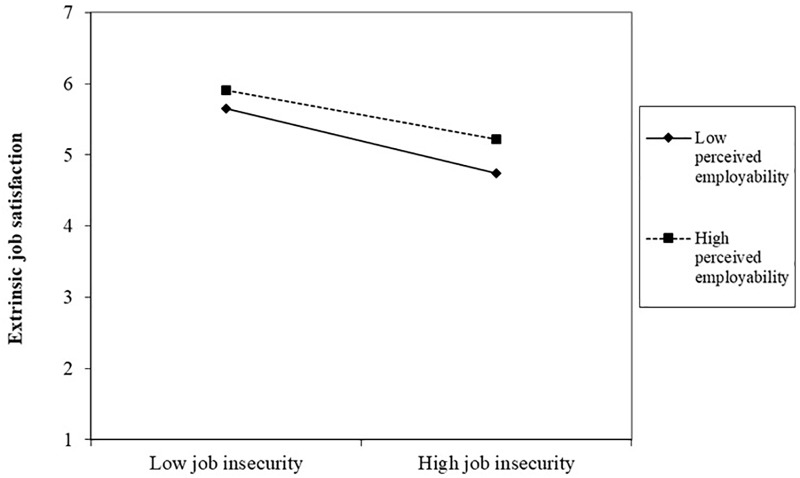
Perceived employability as moderator of the relationship between job insecurity and extrinsic job satisfaction.

**FIGURE 2:**
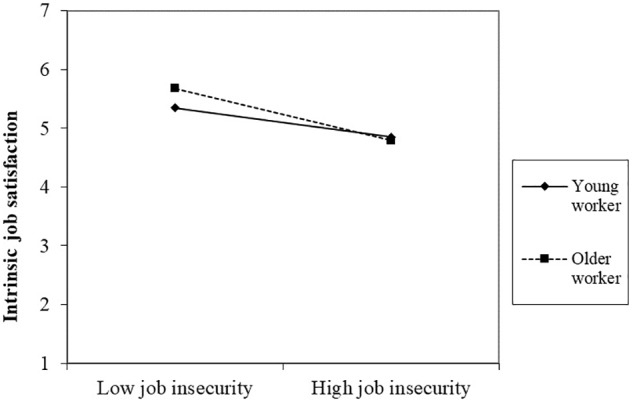
Age as moderator of the relationship between job insecurity and intrinsic job satisfaction.

In hypotheses 2 and 3, perceived employability and age was expected to moderate the relationships between job insecurity and intrinsic and extrinsic job satisfaction. The results obtained at step 3 of the hierarchical multiple regression indicated that interactions for job insecurity and perceived employability were significantly related to extrinsic job satisfaction (β = 0.06; *p* < 0.05), but not intrinsic job satisfaction (β = -0.01; *p* > 0.05). Furthermore, the interaction between job insecurity and age was significantly related to intrinsic job satisfaction (β = -0.08; *p* < 0.05), but not extrinsic job satisfaction (β = -0.03; *p* > 0.05). These interactions above, as well as the three-way interactions, significantly increased the explained variance of intrinsic (Δ*R*^2^= 0.007, *p* < 0.05) and extrinsic job satisfaction (Δ*R*^2^= 0.007, *p* < 0.05). To clarify these results, both interactions were graphically illustrated by plotting moderator variables (perceived employability in Figure 1, and age in Figure 2) at 1 SD below the mean and the other at 1 SD above the mean. For perceived employability as moderator, slopes were significant for employees with high perceived employability (*t* = -7.714; *p* < 0.001) and low perceived employability (*t* = -10.085; *p* < 0.001). Figure 1 shows how perceived employability buffers the negative relationship between job insecurity and extrinsic job satisfaction. In the case of age as a moderator, slopes were also significant for older (*t* = -8.089; *p* < 0.001) and younger employees (*t* = -4.509; *p* < 0.001). Figure 2 shows how the negative effect of job insecurity on intrinsic job satisfaction is weaker in younger than older workers. However, the absolute levels seem to be similar. Thus, these results provide support for the hypotheses 2b and 3a, but not for hypotheses 2a and 3b.

Hypothesis 4 stated that the combination of perceived employability and age would moderate the relationship between job insecurity and extrinsic and intrinsic job satisfaction. In this case, after the control variables, independent variable effects, and interactions among independent variables, results showed that the three-way interaction significantly increased the explained variance of intrinsic job satisfaction (Δ*R*^2^= 0.004, *p* < 0.05), but not extrinsic job satisfaction (Δ*R*^2^= 0.002; *p* > 0.05); in total, the proposed model explained 12.9% of intrinsic job satisfaction variance and 19.4% of extrinsic job satisfaction. Regression analysis showed significant three-way interactions for intrinsic job satisfaction (β = -0.07; *p* < 0.05). Therefore, these results support hypothesis 4a, but not hypothesis 4b.

**FIGURE 3:**
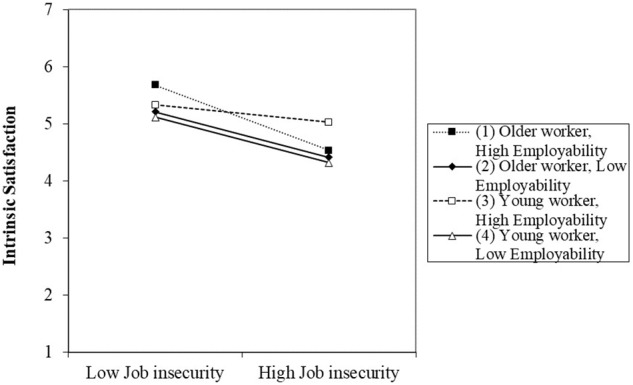
Interaction between job insecurity, perceived employability, and age in predicting intrinsic job satisfaction.

The results of the significant three-way interaction for intrinsic job satisfaction is plotted in Figure 3, which graphically depicts interaction effects among job insecurity, perceived employability, and age on extrinsic and intrinsic job satisfaction. Slopes were significant for younger workers with high perceived employability (*t* = -8.404; *p* < 0.05), younger workers with low perceived employability (*t* = -12.017; *p* < 0.05), older workers with low perceived employability (*t* = -8.310; *p* < 0.05), and older workers with high perceived employability (*t* = -8.855; *p* < 0.05). *t*-Test results of comparisons among slopes indicated that the slope for younger workers with high perceived employability is significant, in contrast to that of older workers with high (*t* = -3.835; *p* < 0.05) and low perceived employability (*t* = -2.246; *p* < 0.05), and that of younger workers with low perceived employability (*t* = 2.702; *p* < 0.05). In addition, the slope of older workers with high perceived employability significantly differs from older workers with low perceived employability (*t* = -2.064; *p* < 0.05). These results indicate that job insecurity negatively affects intrinsic job satisfaction, though the effect is weaker in young workers with high perceived employability than for older workers with high and low perceived employability and for younger workers with low perceived employability. In addition, this effect is weaker in older workers with low perceived employability over those with high perceived employability.

## Discussion

The present study investigated how combinations of age and perceived employability affect the relationship among job insecurity and intrinsic and extrinsic job satisfaction. As expected, job insecurity was negatively related to both job satisfaction dimensions (extrinsic and intrinsic). In the field of job insecurity, these results replicate previous findings on its negative effects on employee attitudes, behaviors, and health (Sverke et al., 2002; Ito and Brotheridge, 2007; Cheng and Chan, 2008; De Cuyper et al., 2009; Silla et al., 2009; Sora et al., 2010; König et al., 2011; Debus et al., 2012; Peiró et al., 2012; Jiang and Probst, 2016). In the specific area of studying differentiated extrinsic and intrinsic job satisfaction, our results also partially align with the findings in Buitendach and De Witte (2005), who found that job insecurity negatively affects extrinsic, but not intrinsic, job satisfaction.

Results also show that perceived employability may be relevant in buffering negative job insecurity consequences for extrinsic, but not intrinsic, job satisfaction; previous studies suggested that perceived employability acts as a buffer mechanism between job insecurity and attitudes (Forrier and Sels, 2003; Fugate et al., 2004; De Cuyper et al., 2008; Silla et al., 2009). The findings in this paper, however, provide more specific information about the consequences of job insecurity on job satisfaction: the present study defines job insecurity as the possibility of losing a job, income, and social status related to the job; as such, it clearly impacts extrinsic job satisfaction. In less stable scenarios, this effect is more significant, as observed in this study. It should be noted that the relationships between intrinsic aspects of job satisfaction and job insecurity, though present, did not pass the significance threshold. This may be due to a job preservation motivation mechanism (Shoss, 2017), in which insecure workers are motivated to act in ways that they believe can preserve their jobs, such as making extra efforts to work harder, aiming to be noticed and valued within their jobs. This can have, as a consequence, an increase in intrinsic satisfaction. It could also be a coping strategy, as Shoss (2017) mentioned, because it allows people to regain control over their work: what had been perceived as lost due to job insecurity results in positive outcomes, which leads to higher amounts of intrinsic satisfaction. It is well known in stress theory that people use three types of coping strategies: emotional-focused; avoidance-focused; and problem-focused. Richter et al. (2013) found that emotional-focused and problem-focused were partial moderators of the relationship between job insecurity and job satisfaction. For those oriented toward problem-focused coping strategies, positive outcomes occur when stressors are perceived as controlled (Richter et al., 2013). Therefore, increases in perceived employability can be understood as a problem-focused coping strategy and, in consequence, a resource all workers (but especially older workers) can use to deal with growing job insecurity.

Age was observed to buffer the consequences of job insecurity only in regards to intrinsic satisfaction. As expected, older workers are more vulnerable to job insecurity (Greenhalgh and Rosenblatt, 1984; Cheng and Chan, 2008; Claes and Van De Ven, 2008), which highlights the need to promote interventions. The impact of age on intrinsic (but not extrinsic) satisfaction may be due to older workers being less oriented to extrinsic goals and more oriented to intrinsic ones (Kooij et al., 2011; Truxillo et al., 2012), or since older workers tend to have perceptions of time limitations, as Ng and Feldman (2008). Age changes motivation toward giving more importance to intrinsic aspects of work (Kanfer and Ackerman, 2004), as supported by several studies (Truxillo et al., 2012; Truxillo et al., 2015) and meta-analyses (Kooij et al., 2011).

Regarding three-way interactions, results show that age plays an important role in employees with high perceived employability; however, it has no effect on employees with low perceived employability. In addition, younger workers with a high perceived employability suffer less than do older employees with high perceived employability in terms of intrinsic job satisfaction. Claes and Van De Ven (2008) found similar results for age moderating the relationship between job insecurity and job satisfaction. It seems that the threat of unemployment might impact older employees more deeply because it entails a threat to their career, especially as they approach retirement; for younger employees, in contrast, a job change has fewer implications, and even less for those who perceive themselves as highly employable. Zacher and Frese (2009) found that age was negatively related to perceived opportunities at work, though this effect was reduced when job complexity and control were higher. Therefore, self-perceptions of employability in older workers can be utilized as a resource to cope with low or moderate job insecurity; for higher job insecurity and the perception of less time to recover, however, there will be more negative impact on intrinsic satisfaction. In that sense, the interactions observed in this research imply that protecting effects of high perceived employability can be neutralized in older workers who perceive the threat of losing their jobs. It stands to reason that other factors could trigger job insecurity perceptions, such as social protection systems, ageism and age stereotypes, economic conditions, and so on; this suggests further study is needed in the context of Chile and countries in Latin America.

Between job insecurity and extrinsic satisfaction, only employability, not age, had a moderating effect. This may be related to extrinsic aspects like fear of losing job stability or short-term income, which are insecurities for both younger and older workers; regardless of age, job insecurity affected less employable people more. For intrinsic satisfaction, younger workers feel less threatened by job insecurity when they perceive themselves as more employable.

The empirical evidence yielded in this study has theoretical implications for stress theories. According to the job-demand resources model (Demerouti et al., 2001) and Karasek’s job-demand control model, job insecurity is viewed as a stressor or demand, while employability is a resource to give employees a sense of control for coping with this demand (Silla et al., 2009). However, our results show that perceived employability as a resource acts differently depending on age. For younger workers, perceived employability acts a buffer mechanism, as expected; in the case of older workers, however, perceived employability increases the negative relationship between job insecurity and intrinsic job satisfaction. This highlights the fact that perceived employability does not replace job security – indeed, in the case of older workers, it also fails to reduce the unfavorable consequences of job insecurity. Next, the psychological contract point of view assumes that these processes are static and apply equally to all employees (Bal et al., 2008). Our results show that the consequences of job insecurity on job satisfaction or contract breach differ depending on employee career stage or lifespan.

From a practical perspective, the results are especially relevant for those at the middle or end of their professional careers – since older people are seen by the environment as less employable, they begin to experience greater job insecurity due to prejudices and stereotypes that influence policies and practices in organizations. Managers who concentrate on decreasing the level of perceived job insecurity among employees can expect increased levels of intrinsic and extrinsic job satisfaction. Clarity, transparency, and opportunity in potentially threatening messages are crucial to communicate change processes. In addition, since employability is a protector against the negative effects of job insecurity, organizations and workers can engage in career interventions oriented to plan careers and enhance employability. Interventions for older workers must include career and retirement preparation in order to improve control and other positive resources. Gaines et al. (2018) suggest that retirement-related concerns may be alleviated by developing and implementing strategies for workers to cope with job insecurity, so organizations should manage and develop older worker careers by focusing on intrinsic satisfaction, their most valued aspect.

The present study has some limitations. First, the cross-sectional design does not allow for a cause-effect relationship between variables to be established. Despite this limitation, the findings are congruent with previous literature. Nevertheless, testing hypotheses using a longitudinal design is recommended for future research. A second limitation concerns the risk of common method variance due to using self-reported data. However, correlations among study variables differ in size and, as published studies show, “using a self-report methodology is no guarantee for finding significant results, even with very large samples” (Spector, 2006, p. 224). Finally, although some critical external factors that might have affected our analyses were controlled for, other unmeasured variables may have influenced our results (for example, the type of contract or labor sector).

Future research might expand the aim of the present study. First, relationships might be tested using other employability constructs, such as the dispositional model proposed by Fugate et al. (2004); or the competence model, by Van der Heijde and Van der Heijden (2006). Furthermore, although employability was here studied as a mechanism to cope with job insecurity perceptions, some studies have suggested that employability may increase deviant behaviors and intentions to leave (De Cuyper et al., 2009; Huang et al., 2017). Therefore, future studies should test the role of age on such negative aspects of employability. Second, this model might be extended to examine job insecurity as a collective construct (Sora et al., 2013). Third, research might compare whether effects differ according to quantitative versus qualitative job insecurity (Shoss, 2017).

In sum, our study shows the advantages of perceived employability for young employees in coping with job insecurity. In addition, it demonstrates the necessity to intervene in how older workers perceive their employability, which is not enough to protect them when facing the decision to continue working. Especially when external factors surpass personal factors, as observed, we cannot rely on workers employability as the only key resource in promoting longer careers in times when job insecurity is a permanent and global phenomenon.

## Ethics Statement

This study was carried out in accordance with the recommendations of Adolfo Ibáñez University ethics committee with written informed consent from all subjects. All subjects gave written informed consent in accordance with the Declaration of Helsinki. The protocol was approved by the Adolfo Ibáñez University Ethics Committee.

## Author Contributions

JY conceptualized, formally analyzed, performed the methodology, supervised, visualized, edited, and wrote the review. MB conceptualized, visualized, edited, and wrote the review. LC, CM, and GC edited and wrote the review.

## Conflict of Interest Statement

The authors declare that the research was conducted in the absence of any commercial or financial relationships that could be construed as a potential conflict of interest.

## Footnotes

1 The national classification system of the socioeconomic status in Chile consists of six levels depending on the per capita income by household. In 2017, the current calculating formula replaced the previous one, which was based on the European Society for Opinion and Marketing Research. Each of the six groups has a letter or a combination of letters and numbers assigned (AB, C1a, C1b, C2, C3, and D), which are distributed by descending order, where the first has the highest income, and so on.

## References

[B1] Ahmed A. Andersson L. Hammarstedt M. (2012). Does age matter for employability? A field experiment on ageism in the Swedish labour market. *Appl. Econ. Lett.* 19 403–406. 10.1080/13504851.2011.581199

[B2] Aiken L. S. West S. G. (1991). *Multiple Regression: Testing and Interpreting Interactions.* Thousands Oaks, CA: Sage Publications.

[B3] Andreev K. Kantorová V. Bongaarts J. (2013). *Demographics Components of Future Population Growth.* Report No. 2013/3. New York, NY: United Nations Department of Economic and Social Affairs.

[B4] Aybas M. Elmas S. Dündar G. (2015). Job insecurity and burnout: the moderating role of employability. *Eur. J. Bus. Manag.* 7 195–203.

[B5] Bakker A. Demerouti E. (2013). La teoría de las demandas y los recursos laborales. *Rev. Psicol. Trabajo Organ.* 29 107–115. 10.5093/tr2013a16

[B6] Bakker A. B. Demerouti E. (2007). The job demands-resources model: state of the art. *J. Manag. Psychol.* 22 309–328. 10.1108/02683940710733115

[B7] Bal A. C. Reiss A. E. B. Rudolph C. W. Baltes B. B. (2011). Examining positive and negative perceptions of older workers: a meta-analysis. *J. Gerontol. Series B Psychol. Sci. Soc. Sci.* 66 687–698. 10.1093/geronb/gbr056 21719634

[B8] Bal P. M. De Lange A. H. Jansen P. G. W. Van Der Velde M. E. G. (2008). Psychological contract breach and job attitudes: a meta-analysis of age as a moderator. *J. Vocat. Behav.* 72 143–158. 10.1016/j.jvb.2007.10.005

[B9] Benach J. Vives A. Amable M. Vanroelen C. Tarafa G. Muntaner C. (2014). Precarious employment: understanding an emerging social determinant of health. *Annu. Rev. Public Health* 35 229–253. 10.1146/annurev-publhealth-032013-182500 24641559

[B10] Berntson E. (2008). *Employability Perceptions: Nature, Determinants, and Implications for Health and Well-Being.* Doctoral dissertation, Stockholm University, Stockholm.

[B11] Berntson E. Marklund S. (2007). The relationship between perceived employability and subsequent health. *Work Stress* 21 279–292. 10.1080/02678370701659215

[B12] Berntson E. Näswall K. Sverke M. (2010). The moderating role of employability in the association between job insecurity and exit, voice, loyalty and neglect. *Econ. Ind. Democracy* 31 215–230. 10.1177/0143831X09358374

[B13] Berntson E. Sverke M. Marklund S. (2006). Predicting perceived employability: human capital or labour market opportunities? *Econ. Ind. Democracy* 27 223–244. 10.1177/0143831x06063098

[B14] Böttcher K. Albrecht A. G. Venz L. Felfe J. (2018). Protecting older workers’ employability: a survey study of the role of transformational leadership. *German J. Hum. Res. Manag.* 32 120–148. 10.1177/2397002218763001

[B15] Buitendach J. De Witte H. (2005). Job insecurity, extrinsic and intrinsic job satisfaction and affective organisational commitment of maintenance workers in a parastatal. *S. Afr. J. Bus. Manag.* 36 27–37.

[B16] Cassar L. (2010). *Revisiting Informality: Evidence From Employment Characteristics and Job Satisfaction in Chile. OPHI Working Paper 41.* Oxford: University of Oxford.

[B17] Cheng G. H. L. Chan D. K. S. (2008). Who suffers more from job insecurity? A meta-analytic review. *Appl. Psychol.* 57 272–303. 10.1111/j.1464-0597.2007.00312.x

[B18] Claes R. Van De Ven B. (2008). Determinants of older and younger workers’ job satisfaction and organisational commitment in the contrasting labour markets of Belgium and Sweden. *Ageing Soc.* 28 1093–1112. 10.1017/s0144686x08007423

[B19] Clark A. Oswald A. Warr P. (1996). Is job satisfaction U-shaped in age? *J. Occup. Organ. Psychol.* 69 57–81. 10.1111/j.2044-8325.1996.tb00600.x

[B20] Dawson J. F. Richter A. W. (2006). Probing three-way interactions in moderated multiple regression: development and application of a slope difference test. *J. Appl. Psychol.* 91 917–926. 10.1037/0021-9010.91.4.917 16834514

[B21] De Cuyper N. Bernhard-Oettel C. Berntson E. De Witte H. Alarco B. (2008). Employability and employees’ well-being: mediation by job insecurity. *J. Appl. Psychol.* 57 488–509. 10.1111/j.1464-0597.2008.00332.x

[B22] De Cuyper N. De Witte H. (2006). The impact of job insecurity and contract type on attitudes, well-being and behavioural reports: a psychological contract perspective. *J. Occupat. Organ. Psychol.* 79 395–409. 10.1348/096317905x53660

[B23] De Cuyper N. Mäkikangas A. Kinnunen U. Mauno S. Witte H. D. (2012). Cross-lagged associations between perceived external employability, job insecurity, and exhaustion: testing gain and loss spirals according to the conservation of resources theory. *J. Organ. Behav.* 33 770–788. 10.1002/job.1800

[B24] De Cuyper N. Notelaers G. De Witte H. (2009). Job insecurity and employability in fixed-term contractors, agency workers, and permanent workers: associations with job satisfaction and affective organizational commitment. *J. Occupat. Health Psychol.* 14 193–205. 10.1037/a0014603 19331480

[B25] De Cuyper N. Piccoli B. Fontinha R. De Witte H. (2018). Job insecurity, employability and satisfaction among temporary and permanent employees in post-crisis Europe. *Econ. Ind. Democracy* 10.1177/0143831X18804655

[B26] De Cuyper N. Sulea C. Philippaers K. Fischmann G. Iliescu D. De Witte H. (2014). Perceived employability and performance: moderation by felt job insecurity. *Pers. Rev.* 43 536–552. 10.1108/PR-03-2013-0050

[B27] De Witte H. (1992). *Langdurig Werklozen: Tussen Optimisten en Teruggetrokkenen [The Long-Term Unemployed: Between Optimism and Resignation] (in Dutch).* Leuven: Hoger Instituut van de Arbeid.

[B28] De Witte H. (1999). Job insecurity and psychological well-being: review of the literature and exploration of some unresolved issues. *Eur. J. Work Organ. Psychol.* 8 155–177. 10.1080/135943299398302

[B29] De Witte H. (2000). “Arbeidsethos en jobonzekerheid: meting en gevolgen voor welzijn, tevredenheid en inzet op het werk [Work ethic and job insecurity: assessment and consequences for wellbeing, satisfaction and performance at work],” in *Van Groep Naar Gemeenschap [From Group to Community]*, eds Bouwen R. De Witte K. De Witte H. Taillieu T. (Leuven: Garant), 325–350.

[B30] De Witte H. De Cuyper N. (2015). “Job insecurity and employability,” in *Wiley Encyclopedia of Management*, eds Cooper C. L. Guest D. E. Needle D. J. (Hoboken, NJ: Wiley).

[B31] De Witte H. Vander Elst T. De Cuyper N. (2015). “Job insecurity, health and well-being,” in *Sustainable Working Lives: Managing Work Transitions and Health Throughout the Life Course*, eds Vuori J. Blonk R. Price R. H. (New York, NY: Springer), 109–128.

[B32] Debus M. E. Probst T. M. König C. J. Kleinmann M. (2012). Catch me if I fall! Enacted uncertainty avoidance and the social safety net as country-level moderators in the job insecurity–job attitudes link. *J. Appl. Psychol.* 97:690. 10.1037/a0027832 22448808

[B33] Demerouti E. Bakker A. Nachreiner F. Schaufeli W. (2001). The job demand resources model of burnout. *J. Appl. Psychol.* 86 499–512. 10.1037/0021-9010.86.3.499 11419809

[B34] Drabe D. Hauff S. Richter N. F. (2015). Job satisfaction in aging workforces: an analysis of the USA, Japan and Germany. *Int. J. Hum. Res. Manag.* 26 783–805. 10.1080/09585192.2014.939101

[B35] Emanuel F. Molino M. Lo Presti A. Spagnoli P. Ghislieri C. (2018). A crossover study from a gender perspective: the relationship between job insecurity, job satisfaction, and partners’ family life satisfaction. *Front. Psychol.* 9:1481. 10.3389/fpsyg.2018.01481 30158890 PMC6103470

[B36] Finkelstein L. M. King E. B. Voyles E. C. (2015). Age metastereotyping and cross-age workplace interactions: a meta view of age stereotypes at work. *Work Aging Retir.* 1 26–40. 10.1093/workar/wau002

[B37] Finkelstein L. M. Ryan K. M. King E. B. (2013). What do the young (old) people think of me? Content and accuracy of age-based metastereotypes. *Eur. J. Work Organ. Psychol.* 22 633–657. 10.1080/1359432x.2012.673279

[B38] Flecker J. Meil P. Pollert A. (1998). The sexual division of labour in process manufacturing: economic restructuring, training and women’s work. *Eur. J. Ind. Relat.* 4 7–34. 10.1177/095968019841002

[B39] Forrier A. Sels L. (2003). The concept employability: a complex mosaic. *Int. J. Hum. Res. Dev. Manag.* 3 102–124. 10.1504/ijhrdm.2003.002414

[B40] Froehlich D. Beausaert S. Segers M. (2015). Age, employability and the role of learning activities and their motivational antecedents: a conceptual model. *Int. J. Hum. Res. Manag.* 26 2087–2101. 10.1080/09585192.2014.971846

[B41] Fugate M. Kinicki A. J. Ashforth E. A. (2004). Employability: a psycho-social construct, its dimensions, and applications. *J. Vocat. Behav.* 65 14–38. 10.1016/j.jvb.2003.10.005

[B42] Fullerton A. S. Wallace M. (2007). Traversing the flexible turn: US workers’ perceptions of job security, 1977–2002. *Soc. Sci. Res.* 36 201–221. 10.1016/j.ssresearch.2005.09.005

[B43] Gaines B. Dugan A. Cherniack M. (2018). The relationship between retirement expectations and job insecurity among the aging US workforce. *Innov. Aging* 2:1005 10.1093/geroni/igy031.3714

[B44] Gamboa J. P. Gracia F. Ripoll P. Peiró J. M. (2009). Employability and personal initiative as antecedents of job satisfaction. *Span. J. Psychol.* 12 632–640. 10.1017/s1138741600001992 19899663

[B45] García-Montalvo J. Peiró J. M. Soro-Bonmatí A. (2003). *Capital Humano. Observatorio de la Inserción Laboral de Los Jóvenes: 1996-2002.* Valencia: Bancaja-Ivie.

[B46] Green F. (2011). Unpacking the misery multiplier: How employability modifies the impacts of unemployment and job insecurity on life satisfaction and mental health. *J. Health Econ.* 30 265–276. 10.1016/j.jhealeco.2010.12.005 21236508

[B47] Greenhalgh L. Rosenblatt Z. (1984). Job insecurity: toward conceptual clarity. *Acad. Manag. Rev.* 9 438–448. 10.5465/amr.1984.4279673

[B48] Guglielmi D. Avanzi L. Chiesa R. Mariani M. G. Bruni I. Depolo M. (2016). Positive aging in demanding workplaces: the gain cycle between job satisfaction and work engagement. *Front. Psychol.* 7:1224. 10.3389/fpsyg.2016.01224 27574514 PMC4983551

[B49] Hillage J. Pollard E. (1998). *Employability: Developing a Framework for Policy Analysis.* Report No: RR85. London: Department for Education and Employment.

[B50] Hirschfeld R. (2000). Does revising the intrinsic and extrinsic subscales of the minnesota satisfaction questionnaire short form make a difference? *Educ. Psychol. Meas.* 60 255–270. 10.1177/00131640021970493

[B51] Huang G. H. Wellman N. Ashford S. J. Lee C. Wang L. (2017). Deviance and exit: the organizational costs of job insecurity and moral disengagement. *J. Appl. Psychol.* 102 26–42. 10.1037/apl0000158 27618406

[B52] Ito J. K. Brotheridge C. M. (2007). Resources, coping strategies, and emotional exhaustion: a conservation of resources perspective. *J. Vocat. Behav.* 63 490–509. 10.1016/s0001-8791(02)00033-7

[B53] Jiang L. Probst T. M. (2016). A multilevel examination of affective job insecurity climate on safety outcomes. *J. Occupat. Health Psychol.* 21:366. 10.1037/ocp0000014 26594845

[B54] Karasek R. A. Jr. (1979). Job demands, job control and mental strain: implications for job redesign. *Admin. Sci. Quart.* 24 285–308.

[B55] Kanfer R. Ackerman P. L. (2004). Aging, work motivation, and adult development. *Acad. Manag. Rev.* 29 440–458.

[B56] Keim A. C. Landis R. S. Pierce C. A. Earnest D. R. (2014). Why do employees worry about their jobs? A meta-analytic review of predictors of job insecurity. *J. Occupat. Health Psychol.* 19 269–290. 10.1037/a0036743 24796228

[B57] Kinnunen U. Mauno S. Nätti J. Happonen M. (2000). Organizational antecedents and outcomes of job insecurity: a longitudinal study in three organizations in Finland. *J. Organ. Behav.* 21 443–459.

[B58] Kluytmans F. Ott M. (1999). Management of employability in the Netherlands. *Eur. J. Work Organ. Psychol.* 8 261–272.

[B59] König C. J. Probst T. M. Staffen S. Graso M. (2011). A Swiss–US comparison of the correlates of job insecurity. *Appl. Psychol.* 60 141–159. 10.1111/j.1464-0597.2010.00430.x

[B60] Kooij D. De Lange A. Jansen P. Kanfer R. Dikkers J. S. (2011). Age and work-related motives: results of a meta-analysis. *J. Organ. Behav.* 32 197–225. 10.1002/job.665

[B61] Kornadt A. E. Rothermund K. (2011). Contexts of aging: assessing evaluative age stereotypes in different life domains. *J. Gerontol. B Psychol. Sci. Soc. Sci.* 66 547–556. 10.1093/geronb/gbr036 21571702

[B62] Lazarus R. S. Folkman S. (1984). *Stress, Appraisal, and Coping.* New York, NY: Springer.

[B63] Lee C. Huang G. H. Ashford S. J. (2018). Job insecurity and the changing workplace: recent developments and the future trends in job insecurity research. *Ann. Rev. Organ. Psychol. Organ. Behav.* 5 335–359. 10.1146/annurev-orgpsych-032117-104651

[B64] Lu L. Lin H. Y. Lu C. (2015). The moderating role of intrinsic work value orientation on thedual-process of job demands and resources among Chinese employees. *Int. J. Workplace Mental Health* 8 78–91. 10.1108/ijwhm-11-2013-0045

[B65] Masdonati J. Schreiber M. Marcionetti J. Rossier J. (2019). Decent work in Switzerland: context, conceptualization, and assessment. *J. Vocat. Behav.* 110 12–27. 10.1016/j.jvb.2018.11.004

[B66] Mauno S. Kinnunen U. (1999). Job insecurity and well-being: a longitudinal study among male and female employees in Finland. *Community Work Fam.* 2 147–171. 10.1080/13668809908413938

[B67] McArdle S. Waters L. Briscoe J. P. Hall D. T. (2007). Employability during unemployment: adaptability, career identity and human and social capital. *J. Vocat. Behav.* 71 247–264. 10.1016/j.jvb.2007.06.003

[B68] Mohr G. B. (2000). The changing significance of different stressors after the announcement of bankruptcy: a longitudinal investigation with special emphasis on job insecurity. *J. Organ. Behav.* 21 337–359.

[B69] Näswall K. De Witte H. (2003). Who feels insecure in Europe? Predicting job insecurity from background variables. *Econ. Ind. Democracy* 24 189–215. 10.1177/0143831x03024002003

[B70] Ng T. W. H. Feldman D. C. (2008). The relationship of age to ten dimensions of job performance. *J. Appl. Psychol.* 93 392–423. 10.1037/0021-9010.93.2.392 18361640

[B71] Ng T. W. H. Feldman D. C. (2010). The relationships of age with job attitudes: a meta-analysis. *Pers. Psychol.* 63 677–718. 10.1111/j.1744-6570.2010.01184.x

[B72] Oldham G. R. Hackman J. R. (2010). Not what it was and not what it will be: the future of job design research. *J. Organ. Behav.* 31 463–479. 10.1002/job.678

[B73] Owuamalam C. K. Zagefka H. (2014). On the psychological barriers to the workplace: when and why metastereotyping undermines employability beliefs of women and ethnic minorities. *Cult. Div. Ethnic Minor. Psychol.* 20 521–528. 10.1037/a0037645 25313432 PMC4196751

[B74] Peeters E. R. De Cuyper N. De Witte H. (2016). Too employable to feel well? Curvilinear relationship between perceived employability and employee optimal functioning. *Psihologia Resurselor Umane* 14 35–44.

[B75] Peiró J. M. Sora B. Caballer A. (2012). Job insecurity in the younger Spanish workforce: causes and consequences. *J. Vocat. Behav.* 80 444–453. 10.1016/j.jvb.2011.09.007

[B76] Posthuma R. A. Campion M. A. (2008). Twenty best practices for just employee performance reviews: employers can use a model to achieve performance reviews that increase employee satisfaction, reduce the likelihood of litigation and boost motivation. *Compens. Benefits Rev.* 40 47–55. 10.1177/0886368707312139

[B77] Reisel W. D. Probst T. M. Chia S.-L. Maloles C. M. König C. J. (2010). The effects of job insecurity on job satisfaction, organizational citizenship behavior, deviant behavior, and negative emotions of employees. *Inter. Stud. Manag. Organ.* 40 74–91. 10.2753/IMO0020-8825400105

[B78] Richter N. Naswall K. Cuyper N. D. Witte H. D. Hellgren J. (2013). Coping with job insecurity: exploring effects on perceived health and organizational attitudes. *Career Dev. Int.* 18 484–502. 10.1108/CDI-06-2013-0081

[B79] Roskies E. Louis-Guerin C. (1990). Job insecurity in managers: antecedents and consequences. *J. Organ. Behav.* 11 345–359. 10.1002/job.4030110503

[B80] Rothwell A. Arnold J. (2007). Self-perceived employability: development and validation of a scale. *Pers. Rev.* 36 23–41. 10.1108/00483480710716704

[B81] Schreurs B. De Cuyper N. Van Emmerik I. J. H. Notelaers G. De Witte H. (2011). Job demands and resources and their associations with early retirement intentions through recovery need and work enjoyment. *SA J. Ind. Psychol.* 37 1–11. 10.4102/sajip.v37i2.859

[B82] Shoss M. (2017). Job Insecurity: an integrative review and agenda for future research. *J. Manag.* 43 1911–1939. 10.1177/0149206317691574

[B83] Silla I. De Cuyper N. Gracia F. Peiró J. M. De Witte H. (2009). Job insecurity and well – being: moderation by employability. *J. Happ. Stud.* 10 739–751. 10.1007/s10902-008-9119-0

[B84] Sloane P. J. Williams H. (2000). Job satisfaction, comparison earnings, and gender. *Labour* 14 473–502. 10.1111/1467-9914.00142

[B85] Sora B. Caballer A. Peiró J. M. (2010). The consequences of job insecurity for employees: the moderator role of job dependence. *Int. Labour Rev.* 149 59–72. 10.1111/j.1564-913x.2010.00075.x

[B86] Sora B. De Cuyper N. Caballer A. Peiro J. De Witte H. (2013). Outcomes of job insecurity climate: the role of climate strength. *Appl. Psychol.* 62 382–405. 10.1111/j.1464-0597.2012.00485.x

[B87] Spector P. E. (1997). *Job Satisfaction: Application, Assessment, Causes and Consequences.* Thousand Oaks, CA: Sage.

[B88] Spector P. E. (2006). Method variance in organizational research. Truth or urban legend?. *Organ. Res. Methods* 9 221–232. 10.1177/1094428105284955

[B89] Sverke M. Hellgren J. Näswall K. (2002). No security: a meta-analysis and review of job insecurity and its consequences. *J. Occupat. Health Psychol.* 7 242–264. 10.1037/1076-8998.7.3.242 12148956

[B90] Sverke M. Hellgren J. Näswall K. (2006). *Job Insecurity. A Literature Review.* Stockholm: National Institute for Working Life.

[B91] Sverke M. Låstad L. Hellgren J. Näswall K. Richter A. (2018). “Meta-analysis on job insecurity and its outcomes: investigating cross-sectional and longitudinal associations,” in *Proceedings of the 13th Conference of the European Academy of Occupational Health Psychology: Adapting to Rapid Changes in Today’s Workplace*, (Nottingham: European Academy of Occupational Health Psychology), 219–220.

[B92] Tayfur O. Bayhan P. Metin Z. S. Ozsoy A. Kumul B. (2018). Academics’ responses to job insecurity: the mediating effect of job satisfaction. *Higher Educ. Policy* 10.1057/s41307-018-00129-7

[B93] Truxillo D. M. Cadiz D. A. Rineer J. R. Zaniboni S. Fraccaroli F. (2012). A lifespan perspective on job design: fitting the job and the worker to promote job satisfaction, engagement, and performance. *Organ. Psychol. Rev.* 2 340–360. 10.1177/2041386612454043

[B94] Truxillo D. M. Cadiz D. M. Hammer L. B. (2015). Supporting the aging workforce: a review and recommendations for workplace intervention research. *Annu. Rev. Organ. Psychol. Organ. Behav.* 2 351–381. 10.1146/annurev-orgpsych-032414-111435

[B95] Van der Heijde C. M. Van der Heijden B. (2006). A competence-based and multi-dimensional operalization and measurement of employability. *Hum. Res. Manag.* 45 449–476. 10.1002/hrm.20119

[B96] Van der Heijden B. (2002). Prerequisites to guarantee life-long employability. *Pers. Rev.* 31 44–61. 10.1108/00483480210412418

[B97] Van der Heijden B. Gorgievski M. De Lange A. (2016). Learning at the workplace and sustainable employability: a multi-source model moderated by age. *Eur. J. Work Organ. Psychol.* 25 13–30. 10.1080/1359432x.2015.1007130

[B98] Van Emmerik I. J. H. Schreurs B. De Cuyper N. Jawahar I. M. Peeters M. C. (2012). The route to employability: examining resources and the mediating role of motivation. *Career Dev. Int.* 17 104–119.

[B99] Van Hootegem A. De Witte H. De Cuyper N. Elst T. V. (2018). Job insecurity and the willingness to undertake training: the moderating role of perceived employability. *J. Career Dev.* 10.1177/0894845318763893

[B100] Van Selm M. Van der Heijden B. I. J. M. (2013). Communicating employability enhancement throughout the life-span: a national intervention program aimed at combating age-related stereotypes at the workplace. *Educ. Gerontol.* 39 259–272. 10.1080/03601277.2013.750965

[B101] Vander Elst T. De Witte H. De Cuyper N. (2014). The job insecurity scale: a psychometric evaluation across five European countries. *Eur. J. Work Organ. Psychol.* 23 364–380. 10.1080/1359432x.2012.745989

[B102] Vives A. González F. Molina A. Gray N. (2016). The Chilean ageing workforce: who works into old age and under what employment conditions? *Occupat. Environ. Med.* 73 64–65. 10.1136/oemed-2016-103951.174 29579144

[B103] Vorauer J. D. Main K. J. O’Connell G. B. (1998). How do individuals expect to be viewed by members of lower status groups? Content and implications of meta-stereotypes. *J. Pers. Soc. Psychol.* 75 917–937. 10.1037/0022-3514.75.4.917 9825528

[B104] Weiss D. Sassenberg K. Freund A. M. (2013). When feeling different pays off: how older adults can counteract negative age-related information. *Psychol. Aging* 28 1140–1146. 10.1037/a0033811 23957227

[B105] Wittekind A. Raeder S. Grote G. (2010). A longitudinal study of determinants of perceived employability. *J. Organ. Behav.* 31 566–586. 10.1002/job 27126303

[B106] Wunder C. Schwarze J. (2006). *Income Inequality and Job Satisfaction of Full-Time Employees in Germany, Berlin, and IZA Bonn Discussion Paper No. 2084.* Available at: http://ftp.iza.org/dp2084.pdf (accessed April, 2018).

[B107] Zacher H. Frese M. (2009). Remaining time and opportunities at work: relationships between age, work characteristics, and occupational future time perspective. *Psychol. Aging* 24 487–493. 10.1037/a0015425 19485664

